# Dietary Supplementation with Different Types of Potassium and Magnesium during Late Gestation and Lactation Modulates the Reproductive Performance, Antioxidant Capacity, and Immune Function of Sows

**DOI:** 10.3390/ani13132183

**Published:** 2023-07-03

**Authors:** Zixi Wei, Lei Xu, Rong Bai, Limin Cui, Huigang Han, Yulong Han, Wenjuan Sun, Yanpin Li, Xianren Jiang, Xilong Li, Yu Pi

**Affiliations:** 1 Key Laboratory of Feed Biotechnology of Ministry of Agriculture and Rural Affairs, Institute of Feed Research, Chinese Academy of Agricultural Sciences, Beijing 100081, China; 13126830289@163.com (Z.W.); xlei0611@163.com (L.X.); rong.bai@wur.nl (R.B.); sunwenjuan@caas.cn (W.S.); liyanpin@caas.cn (Y.L.); jiangxianren@caas.cn (X.J.); 2 Precision Livestock and Nutrition Unit, TERRA Teaching and Research Centre, Gembloux Agro-Bio Tech, University of Liège, 5030 Gembloux, Belgium; 3 Department of Business Economics, Wageningen University, 6700 EW Wageningen, The Netherlands; 4 Qinghai Yuhong Biotechnology Co., Ltd., Haibei 810200, China; cuilimin0526@163.com; 5 Shandong Provincial Feed Veterinary Medicine Quality Inspection Center, Shandong Provincial Bureau of Animal Husbandry and Veterinary Medicine, Jinan 250022, China; hanhuigang2023@163.com; 6 Haidu College, Qingdao Agricultural University, Qingdao 265200, China; han1102055427@163.com

**Keywords:** potassium-magnesium sulfate, reproductive performance, antioxidant capacity, immunity, sows

## Abstract

**Simple Summary:**

Potassium and magnesium trace minerals play a critical role in the biological metabolic functions of livestock. This study was undertaken to investigate the effects of dietary supplementation with different types of potassium and magnesium, synthetic (magnesium sulfate and potassium chloride) or natural (potassium-magnesium sulfate), on the reproductive performance, antioxidant capacity, and immunity of sows. We found that dietary supplementation with natural potassium and magnesium during late gestation could enhance sows’ antioxidant capacity and the immunoglobulin A level in colostrum. Compared with the synthetic potassium and magnesium, the natural potassium and magnesium functioned better, reflected by potentially mitigating the incidence of intrauterine growth restriction of piglets and decreasing the concentration of the proinflammatory cytokine level in plasma. These findings have important implications for the application of magnesium and potassium trace minerals in sow production to improve reproductive performance.

**Abstract:**

The objective of this study was to investigate the effects of dietary supplementation with different types of potassium and magnesium on the reproductive performance, antioxidant capacity, and immunity of sows. Forty-five Landrace × Yorkshire sows at the late gestation stage (85 d) were randomly assigned to three groups (n = 15). Sows in the control group (CON), potassium chloride and magnesium sulfate group (PM), and potassium-magnesium sulfate group (PMS) were fed with a basal diet, a basal diet supplemented with magnesium sulfate (0.20%) and potassium chloride (0.15%), or a basal diet supplemented with potassium-magnesium sulfate (0.45%), respectively. The results showed that dietary supplementation with PMS did not yield significant effects on the reproductive performance compared with the CON group (*p* > 0.05). However, it significantly elevated the level of insulin-like growth factor 1 (IGF-1) in plasma and immunoglobulin A (IgA) in colostrum (*p* < 0.05). Furthermore, PMS significantly augmented the activities of catalase (CAT) and superoxide dismutase (SOD) while reducing the levels of malondialdehyde (MDA) in comparison to the CON group (*p* < 0.05). Compared with the PM group, the PMS group significantly reduced the incidence rate of intrauterine growth restriction (IUGR) (*p* < 0.05) and significantly decreased the concentration of the proinflammatory cytokine (TNF-α) level in plasma (*p* < 0.05). These results indicated that dietary supplementation with PMS during late gestation could enhance sows’ antioxidant capacity and the IgA level in colostrum. These findings will provide a theoretical reference for the use of magnesium and potassium in sow production to improve sows’ health.

## 1. Introduction

During pregnancy, especially during late gestation, sows are experiencing progressive oxidative stress because of the significant increase in the fetal and mammary gland growth rates [1,2,3], accompanied by a decrease in antioxidant enzymes in the sows’ blood [4]. Accumulating evidence indicates that maternal oxidative stress is associated with the occurrence of poor pregnancy outcomes, such as fetal death, preeclampsia, preterm birth, and intrauterine growth restriction (IUGR) [5,6]. Thus, relieving maternal oxidative stress during late pregnancy can be an effective strategy to improve sow reproductive performance.

Magnesium (Mg) and potassium (K) are essential major elements for animals and are utilized in the form of cations in intracellular fluid, where they play important roles in many physiological functions [7,8]. A Mg ion is required for adenosine triphosphate (ATP) synthesis and proper functioning of the immune system, and it enhances resistance to stress [9,10]. Previous research has demonstrated that magnesium serves as a cofactor or activator in over 600 enzymatic reactions [11], exerting an extremely broad range of effects on pregnant women [12]. Studies also showed that maternal supplementation of catalase (CAT) enhanced the concentration of Mg in the serum of newborn piglets, indicating a potential correlation between Mg and CAT [13,14]. Mg deficiency during gestation could exert an impact on maternal and fetal fatty acid metabolism, fetal growth, as well as fetal survival in both rodents and humans [15]. Studies have demonstrated that supplementation with Mg increased the plasma immunoglobulin G (IgG) level of sows, prevented constipation [16], and improved reproduction performance by decreasing the mortality rate of newborn piglets [17]. K can regulate cellular osmotic pressure, maintain the acid–base balance in body fluids, and participate in the metabolism of carbohydrates and proteins [7]. The addition of K-containing compounds in drinking water reduced the heat stress of broilers [18]. In addition, it was reported that dietary supplementation with potassium-magnesium sulfate enhanced growth performance, reduced diarrhea incidence, and modulated the antioxidant capacity and intestinal immunity in weaned piglets [19]. However, the effects of the joint use of K and Mg on reproductive performance, antioxidant capacity, and immunity in sows in the gestation period remain unclear.

Therefore, this study was designed to investigate the effect of dietary supplementation with two types of K and Mg: a synthetic potassium and magnesium (MgSO_4_ and KCl), and a natural potassium and magnesium (potassium-magnesium sulfate, K_2_SO_4_.MgSO_4_·4H_2_O), on the reproductive performance, antioxidant capacity, and immunity of sows in the late gestation period.

## 2. Materials and Methods

### 2.1. Animal Ethics

The animal procedures in this experiment were approved by the Institute Animal Care and Use Committee of the Institute of Feed Research of the Chinese Academy of Agricultural Sciences (IFR-CAAS20211205).

### 2.2. Animals and Experimental Designs

**Figure 1:**
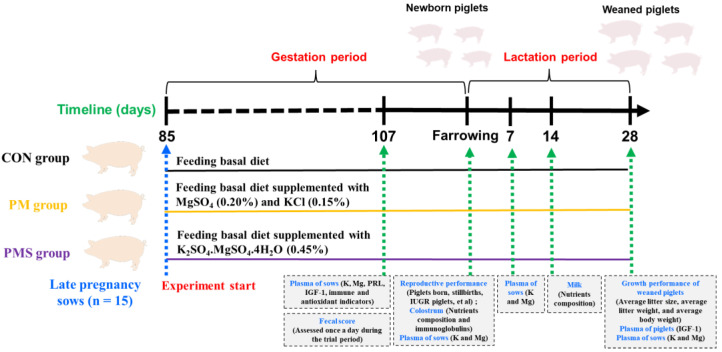
Experimental design and sample collection timeline. PRL, prolactin; IGF-1, insulin-like growth factor 1; IUGR, intrauterine growth restriction.

A total of forty-five healthy crossbred multiparous sows (Landrace × Yorkshire; ages ranged from 2~4 parity; body weight 228.4 ± 21.47 kg; backfat thickness 16.2 ± 1.07 mm) were randomly assigned to 3 groups on day 84 of gestation. The backfat thickness of the sows was measured with an ultrasonic meter (ECM, France), and the measuring position was 6–8 cm from the middle back line of the last rib on the left side of the sows. From day 85 of gestation until the weaning of piglets, sows were allocated to either a control group receiving a corn–soybean meal basal diet (CON group) or one of two treatment groups receiving diets supplemented with a synthetic compound of potassium and magnesium (PM group, basal diet with 0.20% magnesium sulfate and 0.15% potassium chloride) or a natural compound of potassium and magnesium (PMS group, basal diet with 0.45% potassium-magnesium sulfate); the amount of natural or chemical synthetic compound added was determined based on each sow’s nutritional requirements as well as the purity of the compound (Figure 1). The potassium-magnesium sulfate products were obtained from Qinghai Yuhong Biotechnology Co., Ltd. (Haibei, China), which were composed of K_2_SO_4_·MgSO_4_·4H_2_O (K: 21.63%, Mg: 10.86%). As a natural green feed additive, the potassium-magnesium sulfate product was extracted from the potassium-magnesium sulfate ore collected from the Chaerhan Salt Lake. In detail, according to the different solubilities of each salt, the brine of the Qinghai Salt Lake was prepared by the sunning method. The natural brine composition is as follows: NaCl, 20–23%; KCl, 0.4–0.8%; MgSO_4_, 5–7%; MgCl_2_, 4–6%. In natural brine, only NaCl is saturated, so in the first stage of evaporation, the solid component is NaCl. When the brine evaporates to NaCl: 0.8–1%, KCl: 3.3–3.6%, MgSO_4_: 5–6%, MgCl_2_: 23–24%, the crystallized salt combination is KCl·MgCl_2_·6H_2_O (carnallite), NaCl, MgSO_4_·6H_2_O. The obtained solid is added to the appropriate amount of fresh water, maintained at the appropriate temperature, and then the K_2_SO_4_·MgSO_4_·6H_2_O product is obtained.

All diets were formulated to meet the nutrient requirements for sows as specified by the National Research Council (NRC, 2012) [20] (Table 1). During the whole experimental period, sows were housed in individual gestation stalls and fed three times a day (at 7:00, 11:00, and 17:30). From day 85 to farrow, sows received 3.0 kg/day of diets. They were allowed ad libitum access to feed after farrowing until weaning. On day 107 of gestation, sows were moved to the farrowing rooms and housed in individual farrowing crates. All the sows had free access to water.

### 2.3. Data and Sample Collection

During farrowing, the total number of piglets born, including those born alive, stillborn, and mummified, as well as their individual birth weights were meticulously recorded. Piglets were weaned on day 28 and the litter size and body weight were recorded. The fecal score of sows was assessed directly after defecation using the visual qualitative evaluation method: 1 = dry and pellet-shaped; 2 = between dry and normal, soft but firm and well-formed; 3 = soft and well-formed; 4 = between normal and wet, still formed but not firm; and 5 = wet feces, unformed and liquid.

On day 107 of gestation, approximately 7 mL of blood samples from each sow were collected into heparin tubes from the ear vein and immediately placed in an ice box before centrifugation. On day 14 after the birth of piglets, one piglet was randomly taken from each litter, and an approximately 7 mL blood sample was taken from its jugular vein into a heparin tube and immediately placed in an ice box before centrifugation. The blood samples were centrifuged at 3000× *g* under 4 °C for 15 min (Eppendorf centrifuge 5810R, Hamburg, Germany). The plasma samples obtained from the supernatant were subsequently preserved at −20 °C for further analysis of immune and antioxidant indicators.

Colostrum samples were collected manually from all active mammary glands on one side within 1 h of farrowing onset, while milk samples were obtained on day 14 of lactation following intravenous injection of 2.0 mL oxytocin (no. hc00011) (Hangzhou Animal Medicine Factory, Hangzhou, China) via the ear vein. The samples were immediately stored at −20 °C for a further analysis of nutrient components and immunoglobulins.

### 2.4. Chemical Analysis of Diets

The samples of diet were analyzed for dry matter (DM), crude protein (CP) (N × 6.25), calcium, and phosphorous, according to the procedures of the Association of Official Analytical Chemists (2003). The concentrations of potassium and magnesium in the diet were determined using the method of atomic absorption spectroscopy according to the People’s Republic of China National Standard GB/T 18633-2018 and GB/T 13885-92, respectively.

### 2.5. Concentration of Potassium and Magnesium in Plasma Determination

The concentrations of potassium (no. AKBL004M) and magnesium (no. AKBL005M) in plasma were determined using commercial reagent kits according to the manufacturer’s instructions (Beijing Boxbio Science & Technology Co., Ltd., Beijing, China).

### 2.6. Milk Composition Determination

The concentrations of protein, fat, lactose, and solids-not-fat in colostrum and milk samples were determined through analysis after appropriate dilution using an automatic milk analyzer (Milk-Yway-CP2, Beijing, China). The results were expressed as percentages in samples.

### 2.7. Colostrum Immunoglobulin Determination

The levels of immunoglobulin G (IgG) (no. ml002328), immunoglobulin A (IgA) (no. ml087739), and immunoglobulin M (IgM) (no. ml002334) were determined using commercial reagent kits according to the manufacturer’s instructions (Enzyme-linked Biotechnology Co. Ltd., Shanghai, China).

### 2.8. Determination of Plasma Hormones and Immunological and Antioxidant Parameters

The levels of insulin-like growth factor 1 (IGF-1) (ml002344-C), prolactin (PRL) (no. ml08034), interleukin-1β (IL-1β) (no. ml002302), interleukin-6 (IL-6) (no. ml002311), tumor inflammatory factor-α (TNF-α) (no. ml002360), and interleukin-10 (IL-10) (no. ml002319) were determined using commercial reagent kits according to the manufacturer’s instructions (Enzyme-linked Biotechnology Co. Ltd., Shanghai, China). The levels of malondialdehyde (MDA) (no. BC0025) and total antioxidant capacity (T-AOC) (no. BC1315) and the activity of catalase (CAT) (no. BC0205) and superoxide dismutase (SOD) (no. BC0175) were determined using commercial reagent kits according to the manufacturer’s instructions (Beijing Solarbio Science & Technology Co., Ltd., Beijing, China). The experimental design and sample collection timeline described above are shown in Figure 1.

### 2.9. Statistical Analysis

The data were analyzed using a completely randomized design with repeated measures, followed by one-way ANOVA and Tukey’s multiple comparison test in SPSS 20.0 software (SPSS Inc., Chicago, IL, USA). Statistically significant differences were declared at *p* < 0.05.

## 3. Results

### 3.1. Reproductive Performance and the Influence of Fecal Score

The effects of maternal dietary supplementation with PM or PMS on the sows’ reproductive performance and weaned piglets’ growth performance are shown in Table 2 and Table 3. Dietary supplementation with PMS did not yield significant effects on the total number of piglets born, number of live-born piglets, average initial body weight, average weight of litter, number of weaned piglets, average weaning weight, and weaning litter weight compared with the CON group (*p* > 0.05). Additionally, supplementation with PM resulted in an increased incidence of IUGR piglets and coefficient of variation (CV) of within-litter birth weight (*p* < 0.05), while supplementation with PMS showed no significant difference in those compared to the CON group (*p* < 0.05). Compared with the PM group, the PMS group significantly reduced the incidence of IUGR piglets and CV of within-litter birth weight (*p* < 0.05). There was no statistically significant difference observed in the growth performance of piglets within 28 days before weaning after adding either PM or PMS (*p* > 0.05). The fecal score of sows indicated that supplementation with PM or PMS did not result in a significant improvement in constipation throughout the entire trial period (*p* > 0.05) (Table 4).

### 3.2. Hormone, Potassium, and Magnesium Levels in Plasma

**Figure 2:**
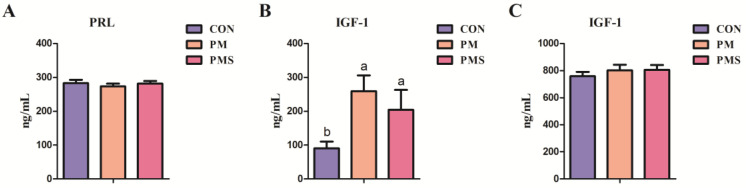
Effects of maternal dietary supplementation with two types of potassium and magnesium on the concentrations of PRL and IGF-1 in plasma of gestation sows or piglets (n = 15): (**A**) plasma PRL of sows; (**B**) plasma IGF-1 of sows; (**C**) plasma IGF-1 of piglets. All data are presented as mean ± SEM. ^a,b^ The values with different superscripts are significantly different at *p* < 0.05. CON: sows were fed with a corn–soybean meal basal diet; PM: sows were fed with a basal diet supplemented with 0.20% magnesium sulfate and 0.15% potassium chloride; PMS: sows were fed with a basal diet supplemented with 0.45% natural potassium-magnesium sulfate. PRL, prolactin; IGF-1, insulin-like growth factor 1.

The effect of maternal dietary supplementation with PM or PMS on the levels of potassium and magnesium in the plasma of sows is shown in Table 5. Overall, compared to the CON group, supplementing magnesium and potassium ions during gestation and lactation increased the levels of K and Mg in sow blood to varying degrees, particularly evident during late gestation and early parturition (day 0 and day 7). The PM and PMS groups showed increased serum potassium levels on day 107 of gestation and day 0 and day 7 of lactation (*p* < 0.05). On day 0 of lactation, the increase in potassium levels induced by PMS was higher than the CON and PM groups; however, no significant difference was detected between the PMS and PM groups. Furthermore, the PMS group exhibited a significantly higher level of potassium on day 7 of lactation compared to the PM group (*p* < 0.05). As for magnesium, PM and PMS supplementation resulted in an increased level of magnesium in the blood on day 7 of lactation (*p* < 0.05). On day 0 of lactation, the level of magnesium in the PMS group was higher than that in the CON group (*p* < 0.05); however, no significant difference was observed between the PMS and PM groups (*p* > 0.05). The effects of maternal dietary supplementation with PMS or PM on the concentration of PRL and IGF-1 in the plasma of sows or piglets are shown in Figure 2. There was no statistically significant difference in the levels of PRL among the three groups on day 107 of gestation (*p* > 0.05) (Figure 2A). In comparison to the CON group, the administration of PMS significantly elevated IGF-1 levels in plasma on day 107 of gestation (*p* < 0.05) (Figure 2B). However, there was no significant difference in IGF-1 levels in the plasma of weaned piglets among the three groups on day 28 (Figure 2C).

### 3.3. The Composition of and Immunoglobulin in Sow Milk

**Figure 3:**
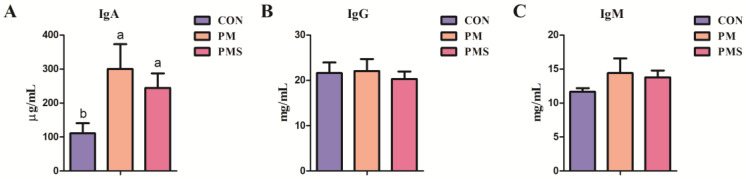
Effects of maternal dietary supplementation with two types of potassium and magnesium on the immunoglobulin in colostrum of sows (n = 15): (**A**) IgA; (**B**) IgG; (**C**) IgM. All data are presented as mean ± SEM. ^a,b^ The values with different superscripts are significant differences at *p* < 0.05. CON: sows were fed with a corn–soybean meal basal diet; PM: sows were fed with a basal diet supplemented with 0.20% magnesium sulfate and 0.15% potassium chloride; PMS: sows were fed with a basal diet supplemented with 0.45% natural potassium-magnesium sulfate. IgA, immunoglobulin A; IgG, immunoglobulin G; IgM, immunoglobulin M.

Table 6 illustrates the effect of dietary supplementation with PM or PMS on the composition of colostrum and ordinary milk. Dietary supplementation with PM or PMS did not exert a significant effect on the contents of protein, fat, lactose, total solids, and solids-not-fat in the colostrum and ordinary milk (*p* > 0.05) (Table 6). The effects of maternal dietary supplementation with different types of potassium and magnesium on the immunoglobulins in the colostrum of sows are shown in Figure 3. Supplementation with PM or PMS in the diet resulted in a significant increase in the IgA level present in colostrum on day 0 of lactation compared to the CON group (*p* < 0.05) (Figure 3A). However, there were no significant changes in IgM and IgG among the three groups (*p* > 0.05) (Figure 3B,C).

### 3.4. Antioxidant Index and Immune Cytokines in Plasma

**Figure 4:**
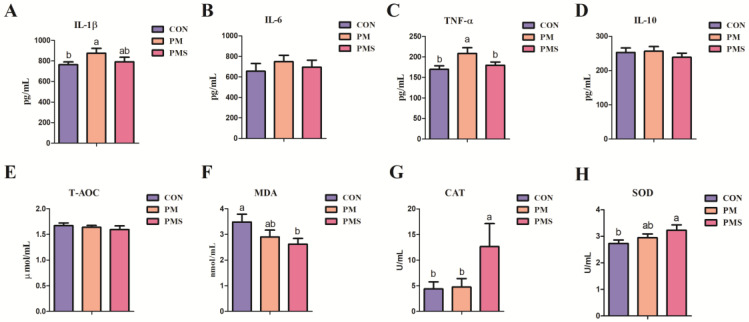
Effects of maternal dietary supplementation with two types of potassium and magnesium on the antioxidant indicators and immune cytokines in plasma of gestation sows (at 107 d) (n = 15): (**A**) IL-1β; (**B**) IL-6; (**C**) TNF-α; (**D**) IL-10; (**E**) T-AOC; (**F**) MDA; (**G**) CAT; (**H**) SOD. All data are presented as mean ± SEM. ^a,b^ The values with different superscripts are significant differences at *p* < 0.05. CON: sows were fed with a corn–soybean meal basal diet; PM: sows were fed with a basal diet supplemented with 0.20% magnesium sulfate and 0.15% potassium chloride; PMS: sows were fed with a basal diet supplemented with 0.45% natural potassium-magnesium sulfate. IL, interleukin; TNF-α, tumor necrosis factor-α; T-AOC, total antioxidant capacity; MDA, malondialdehyde; CAT, catalase; SOD, superoxide dismutase.

The effects of dietary PMS on the plasma antioxidant capacity and immune function in late gestation sows are presented in Figure 4. Dietary supplementation with PMS did not result in any significant alterations in the plasma concentrations of IL-1β, IL-6, TNF-α, and IL-10 compared with the CON group (*p* > 0.05) (Figure 4A–D). However, dietary supplementation with PM increased the concentrations of IL-1β and TNF-α compared to the other two groups (*p* < 0.05) (Figure 4A,C). Moreover, PMS significantly augmented the activities of CAT in comparison to that of the PM and CON groups, and PMS also augmented the activities of SOD while reducing the levels of MDA in comparison to that in the CON group (*p* < 0.05) (Figure 4F–H), whereas PMS did not affect the activities of T-AOC (Figure 4E). In addition, supplementation with PM did not result in any significant differences in the activities of CAT, SOD, MDA, and T-AOC. However, there was a tendency towards increased SOD activity and decreased MDA activity compared to the CON group (Figure 4E–H).

## 4. Discussion

During the final month of gestation, sows undergo a rapid acceleration of fetal growth and development, which coincides with an intensified metabolic state. This heightened metabolic burden often results in systemic oxidative stress for the sows. Sows experiencing oxidative stress may exhibit adverse symptoms, such as reduced feed intake, electrolyte imbalances, and impaired fetal development [21]. Additionally, the milk synthesis during lactation in sows can result in an augmented physical burden, also leading to oxidative stress that negatively impacts milk production and other factors crucial for piglet growth [21]. Previous research has demonstrated the potential for supplemental magnesium to enhance sows’ reproductive performance, with optimal dosages ranging from 0.015% to 0.03% [22]. Hou et al. demonstrated that supplementation with MgSO_4_ at doses of 200, 400, or 600 mg/kg resulted in a linear decrease in both the survival rate and litter weight at weaning, without affecting other parameters related to sow or litter performance. Moreover, dietary supplementation with potassium-magnesium sulfate could enhance growth performance, reduce diarrhea incidence, enhance antioxidant capacity, and modulate intestinal immunity in weaned piglets [19]. Andrea et al. reported that the administration of an injected mineral supplement (Forssman) containing selenium, copper, potassium, and magnesium during summer months was found to effectively restore ovarian function in heat-stressed lactating dairy cows [23]. Although magnesium has been studied in terms of the reproductive performance, immunity, and antioxidant capacity of sows, the joint use of potassium and magnesium is rarely reported in the late gestation period. Therefore, the current study was designed to examine the effect of dietary supplementation with two types of K and Mg compounds, a synthetic potassium and magnesium (MgSO_4_ and KCl) and a natural potassium and magnesium (potassium-magnesium sulfate, K_2_SO_4_·MgSO_4_·4H_2_O), on sow reproductive performance, antioxidant capacity, and immunity in last gestation period.

In the current study, PMS is more effective than PM in reducing the occurrence of mummy and IUGR piglets (Table 1). Trawńska et al. found that dietary supplementation of MgCl_2_·6H_2_O at a dose of 1 g/100 kg BW/d, equivalent to 120 mg pure magnesium, could effectively reduce the mortality rate of the newborn piglets and improve the survival rate and litter weight until 21 days [17]. Katalin et al. also reported that magnesium supplementation had a positive impact on the reproductive performance, specifically in terms of the conception rate and litter size; however, it did not reduce the stillbirth rate. Zang et al. demonstrated that magnesium supplementation for gilts did not have a significant impact on the total number of piglets born, the number of piglets born alive, and the litter size [22]. Their study explained that the less significant improvement of gilts’ reproductive performance compared to multiparous sows may be attributed to the higher magnesium requirement of sows as they age. This study indicated that supplementation of PM or PMS had a positive effect on the reproductive performance of sows and tended to decrease the stillbirth rate more effectively than supplementation with magnesium solely, and PMS was more effective than PM in decreasing the occurrence of mummies and IUGR piglets. This indicated that the natural compound mineral additive of potassium and magnesium could improve the reproductive performance of sows better than synthetic potassium and magnesium. At present, there are some explanations for magnesium improving sow reproductive performance. For example, it may be related to a reduced incidence of constipation. Constipation has been demonstrated to have a negative impact on the reproductive performance of sows. Magnesium supplementation has been successfully used as a laxative to alleviate constipation in sows during both gestation and lactation periods [17]. In this study, it can be found that supplementation with PM or PMS had no significant effect on the fecal score of sows during the whole trial period. This indicated that the improvement of the performance of sows by PM or PMS is not the reason for improving the constipation of sows. Another possible reason is that this result was due to improvements in systemic defense and/or hormonal levels. Therefore, further measurements were conducted to investigate these hypotheses.

Magnesium serves as a crucial cofactor for several enzymes involved in protein and energy metabolism, playing an integral role in numerous biochemical processes such as phosphate activation and carbohydrate metabolism. Potassium plays a vital role in regulating the osmotic pressure of cells, as well as maintaining the acid–base and electrolyte balances of body fluids. Agnès Jamin showed that enrichment of the monopotassium-phosphate formula restored whole-body anabolism in IUGR piglets [24]. Previous studies yielded inconsistent findings regarding the effect of supplementation on serum magnesium levels in swine. Harmon et al. reported that an elevation in dietary magnesium intake resulted in a corresponding increase in serum magnesium levels among pigs [25]. Hou et al. found that the plasma Mg concentrations were significantly increased with the supplementation of MgSO_4_ [16]. However, Svajgr et al. reported that the supplementation of magnesium harmed the serum magnesium levels in growing and finishing pigs [26]. Ashar demonstrated that blood potassium levels usually decreased during pregnancy, and maintaining potassium homeostasis during pregnancy was essential to prevent maternal hypokalemia [27]. In this study, after supplementation with PM or PMS, the plasma Mg and K concentrations were significantly increased in late gestation and early lactation (day 0 and day 7), and the addition of PMS resulted in higher levels of blood potassium and magnesium on day 0 of lactation compared to that of PM. Magnesium in sow diets could facilitate protein synthesis and strengthen protein deposition [16]. This implies that elevated levels of magnesium and potassium may induce alterations in the body’s biochemical processes. The alterations may exert a favorable effect on the reproductive performance of sows, and adding the natural compound mineral additive of potassium and magnesium had a stronger effect on sows than the chemical synthetic compound of potassium and magnesium.

Colostrum and milk are essential for the growth and development of pre-weaning piglets, as the BW gain of piglets depends on the volume and quality of milk obtained from sows. Stockdale reported that MgSO_4_ did not result in any significant effect on milk yield or composition in cows [28]. Hou et al. found that sows supplemented with MgSO_4_ during late gestation exhibited a reduction in colostrum fat percentage while also demonstrating an increase in milk protein content [16]. In this study, it can be found that supplementation with PM to PMS did not significantly affect milk composition; however, there was a tendency to decrease the fat in colostrum, which is consistent with Hou’s finding. One of the explanations was that this tendency may be attributed to a reduction in plasma triglyceride levels. The present study showed that the addition of PM or PMS increased the levels of potassium and magnesium in the blood but did not affect the composition of the milk. This could also account for the lack of variance in the growth performance (average litter size, average litter weight, and average body weight) during a 28-day weaning period. Mg also plays a crucial role in the modulation of cellular immunity and acts as a cofactor for the synthesis of immunoglobulin [29]. This study also measured the amount of immunoglobulin in colostrum (Figure 3). The inclusion of either PM or PMS resulted in a significant increase in the IgA content. Cao et al. demonstrated that the addition of PMS tended to enhance intestinal IgM levels, which could be attributed to an increase in dietary cations that stimulated immunoglobulin expression [19]. The presence of anionic salt in the sow diet is positively correlated with immunoglobulin level, and because the immune system of newborn piglets is not fully developed, it primarily relies on the immunoglobulin present in colostrum to acquire passive immunity. In pigs, almost all milk IgA is locally synthesized within the mammary gland. This study indicated that PMS or PM could stimulate the mammary tissue to synthesize IgA, thereby strengthening the piglets’ immunity.

IGF-1, acting as a promoter of cell mitosis, is crucial for maintaining the levels of proteins involved in cellular differentiation and plays a pivotal role in newborn growth and metabolism [30]. In this study, it can be found that the dietary addition of PMS significantly improved the levels of IGF-1 in the plasma of gestation sows, suggesting that PMS facilitated fetal growth and development in utero through regulating the level of IGF-1, which may be one of the reasons for reducing the occurrence of IUGR and CV of within-litter birth weight. However, further elevated levels of IGF-1 in the plasma of piglets were not observed, implying that elevated IGF-1 in sows was not transmitted to piglets through milk.

Mg plays a crucial role in modulating lymphocyte growth and thus is essential for acquired immunity. In Mg^2+^-deficient animal models, the observable change is inflammation, characterized by elevated levels of pro-inflammatory cytokines such as TNF-α and decreased levels of anti-inflammatory cytokines. Mg deficiency induces a stress response that activates the sympathetic nervous system and hypothalamic–pituitary axis, leading to adipose tissue accumulation and neuropeptide release; subsequently, an immune response ensues followed by inflammatory cascades. Lopez-Baltanas et al. demonstrated that Mg supplementation mitigated inflammation in rats with experimentally induced chronic kidney disease [31]. Cao et al. demonstrated that the dietary supplementation of PMS resulted in a linear reduction in the levels of inflammatory cytokines IL-8 and IL-1β in the jejunum. In this study, it can be found that adding PM or PMS did not effectively reduce the expression of inflammatory cytokines (IL-6, IL-1β, TNF-α, and IL-10) in the plasma of gestation sows; however, supplementation with PM increased the expression of TNF-α and IL-1β. The failure to achieve the desired anti-inflammatory effect may be attributed to the dosage of PM or PMS. However, the results showed that the natural potassium and magnesium (potassium-magnesium sulfate) could prevent the expression of inflammatory cytokines and avoid the occurrence of inflammation better than the synthetic potassium and magnesium (MgSO_4_ and KCl). This indicated that the addition of the natural potassium and magnesium was comparatively milder and more advantageous to animal physiology than the synthetic potassium and magnesium, which needs further investigation.

Sows experience oxidative stress due to a severe metabolic burden, which persists until the weaning period during late gestation [32]. This process is characterized by the accumulation of active oxygen, including hydrogen peroxide (H_2_O_2_) and superoxide (O_2_^−^), which negatively regulates reproductive performance and lactation performance, and excessive levels of reactive oxygen species (ROS) increase the risk of prenatal death [33]. The antioxidative and anti-inflammatory properties of Mg confer additional protection against various pathological conditions. Mg deficiency induces the downregulation of the electron transport chain (ETC) and the upregulation of ROS production. Additionally, magnesium deficiency can inhibit the antioxidant defense system by reducing protein levels of manganese superoxide dismutase (MnSOD) and CAT [34]. Liu reported that Mg supplementation showed antioxidant properties in patients with Mg deficiency. Cao et al. demonstrated that PMS may increase Peptostreptococcaceae abundance, affecting GSH-Px activity [19]. In this study, it can be found that supplementation of PMS significantly increased the antioxidant capacity in the late gestation of sows compared to the other two groups. This suggested that the natural potassium and magnesium was superior to the synthetic potassium and magnesium in terms of antioxidant capacity. However, supplementation with PM did not result in a significant improvement in antioxidant indicators. To a certain extent, this may suggest that supplementation with PMS resulted in a lower incidence of IUGR piglets closely related to its antioxidant effect. This also explained that the natural PMS is superior to the synthetic PM in improving the productive performance of sows.

## 5. Conclusions

Dietary supplementation with potassium-magnesium sulfate during the late gestation and lactation stages could enhance sows’ antioxidant capacity and improve the level of IgA in colostrum to strengthen immunity. Compared with the synthetic potassium and magnesium, the natural potassium-magnesium sulfate could decrease the incidence of IUGR piglets and the level of proinflammatory cytokine (TNF-α) in plasma. Therefore, potassium-magnesium sulfate may be a potential feed additive to improve the productive performance of sows during the late gestation and lactation stages.

## Figures and Tables

**Table 1:** Ingredient composition and nutritional value of the experimental diets (%, as-fed basis).

Items	Gestation			Lactation		
	CON	PM	PMS	CON	PM	PMS
Corn	66.34	66.34	66.34	62.37	62.37	62.37
Soybean meal, 46% CP	15.50	15.50	15.50	18.50	18.50	18.50
Fish meal	-	-	-	3.00	3.00	3.00
Extruded soybean	7.00	7.00	7.00	6.70	6.70	6.70
Wheat bran	8.00	7.65	7.55	5.00	4.65	4.55
Calcium dihydrogen phosphate	0.85	0.85	0.85	0.95	0.95	0.95
Limestone	1.50	1.50	1.50	1.15	1.15	1.15
Soybean oil	-	-	-	1.00	1.00	1.00
Sodium chloride	0.40	0.40	0.40	0.30	0.30	0.30
Sodium bicarbonate	-	-	-	0.10	0.10	0.10
Sodium sulfate	-	-	-	0.10	0.10	0.10
L-Lysine HCl, 98.5%	0.08	0.08	0.08	0.35	0.35	0.35
L-Threonine	-	-	-	0.05	0.05	0.05
Choline chloride, 60%	0.07	0.07	0.07	0.10	0.10	0.10
Phytase	0.01	0.01	0.01	-	-	-
Antioxidant	0.02	0.02	0.02	0.02	0.02	0.02
Vitamin premix ^1^	0.03	0.03	0.03	0.06	0.06	0.06
Mineral premix ^2^	0.20	0.20	0.20	0.25	0.25	0.25
Magnesium sulfate	-	0.20	-	-	0.20	-
Potassium chloride	-	0.15	-	-	0.15	-
Potassium-magnesium sulfate	-	-	0.45	-	-	0.45
Total	100.00	100.00	100.00	100.00	100.00	100.00
Analyzed nutrient levels, %						
Dry matter	87.30	87.50	87.10	86.80	87.70	87.80
Crude protein	15.75	16.19	15.80	19.05	18.63	20.33
Total calcium	0.80	0.76	0.78	0.76	0.78	0.73
Total phosphorus	0.50	0.50	0.50	0.64	0.64	0.64
Total potassium	0.99	1.18	1.46	1.17	1.35	1.31
Total magnesium	0.18	0.21	0.18	0.17	0.22	0.22
Calculated nutrient levels, kcal/kg						
Metabolizable energy	3108	3108	3108	3183	3183	3183
Lysine, %	0.63	0.63	0.63	0.95	0.95	0.95
Methionine + cysteine, %	0.48	0.48	0.48	0.56	0.56	0.56
Threonine, %	0.51	0.51	0.51	0.65	0.65	0.65
Tryptophan, %	0.16	0.16	0.16	0.20	0.20	0.20

^1^ The premix supplied the following vitamins per kilogram of diet: Vitamin A, 25,000 IU; Vitamin D3, 5000 IU; Vitamin E, 50 IU; Vitamin K, 2.5 mg; Biotin, 0.2 mg; Vitamin B1, 1.0 mg; Vitamin B2, 8.0 mg; Vitamin B6, 3.0 mg; Vitamin B12, 0.020 mg; Niacin, 15.0 mg; Pantothenic Acid, 12.5 mg; Folacin, 1.50 mg. ^2^ The premix supplied the following trace minerals per kilogram of diet: Cu, 15 mg; I, 0.3 mg; Mn, 50 mg; Se, 0.3 mg; Fe, 80 mg; Zn, 100 mg.

**Table 2:** Effects of maternal dietary supplementation with two types of potassium and magnesium on the reproductive performance of the sows (n = 15).

Items	Treatments ^3^			SEM	*p*-Value
	CON	PM	PMS		
Piglets born, n	13.77	15.17	13.83	0.600	0.579
Piglets born alive, n	11.31	12.42	12.33	0.636	0.724
Stillbirths, n	2.23	1.58	1.42	0.272	0.439
Mummy, n	0.23	1.17	0.08	0.262	0.196
Live birth rate, %	79.19	81.88	89.29	2.957	0.363
Stillborn rate, %	19.43	10.11	10.25	2.550	0.228
Mummy rate, %	1.39	8.00	0.46	1.838	0.198
Average birth weight, kg	1.52	1.50	1.57	0.035	0.724
Litter birth weight, kg	17.09	19.95	18.32	0.789	0.351
IUGR piglets ^1^, n	0.57 ^b^	1.54 ^a^	0.54 ^b^	0.193	0.055
IUGR piglet rate, %	4.39 ^b^	11.73 ^a^	5.04 ^b^	1.368	0.050
Within-litter birth weight CV ^2^, %	16.23 ^b^	20.28 ^a^	15.16 ^b^	0.937	0.063

^a,b^ The values with different superscripts are significantly different at *p* < 0.05. ^1^ IUGR, intrauterine growth restriction, birth weight < 1.1 kg. ^2^ CV, coefficient of variation. ^3^ CON: sows were fed with a corn–soybean meal basal diet; PM: sows were fed with a basal diet supplemented with 0.20% magnesium sulfate and 0.15% potassium chloride; PMS: sows were fed with a basal diet supplemented with 0.45% natural potassium-magnesium sulfate.

**Table 3:** Effects of maternal dietary supplementation with two types of potassium and magnesium on the growth performance of weaned piglets (at 28 d) (n = 15).

Items	Treatments ^1^			SEM	*p*-Value
	CON	PM	PMS		
Average litter size, n	9.90	10.17	10.82	0.355	0.582
Average litter weight, kg	84.62	87.06	87.33	3.061	0.930
Average body weight, kg	8.65	8.18	8.28	0.225	0.682

^1^ CON: sows were fed with a corn–soybean meal basal diet; PM: sows were fed with a basal diet supplemented with 0.20% magnesium sulfate and 0.15% potassium chloride; PMS: sows were fed with a basal diet supplemented with 0.45% natural potassium-magnesium sulfate.

**Table 4:** Effects of maternal dietary supplementation with two types of potassium and magnesium on the fecal score of sows (n = 15).

Items	Treatments ^1^			SEM	*p*-Value
	CON	PM	PMS		
Gestation stage	1.44	1.60	1.52	0.076	0.754
Lactation stage	1.65	1.93	1.81	0.098	0.598
Whole trial period	1.55	1.78	1.69	0.068	0.471

^1^ CON: sows were fed with a corn–soybean meal basal diet; PM: sows were fed with a basal diet supplemented with 0.20% magnesium sulfate and 0.15% potassium chloride; PMS: sows were fed with a basal diet supplemented with 0.45% natural potassium-magnesium sulfate.

**Table 5:** Effects of maternal dietary supplementation with two types of potassium and magnesium on the concentration of potassium and magnesium in plasma of sows (n = 15).

Items	Treatments ^1^			SEM	*p*-Value
	CON	PM	PMS		
Gestation 107 d, mmol/L					
Potassium	1.88 ^b^	2.54 ^a^	2.35 ^a^	0.112	0.047
Magnesium	3.58	3.8	3.28	0.202	0.613
Lactation 0 d, mmol/L					
Potassium	1.99 ^b^	2.15 ^ab^	2.72 ^a^	0.125	0.040
Magnesium	2.16 ^b^	2.51 ^ab^	2.98 ^a^	0.156	0.089
Lactation 7 d, mmol/L					
Potassium	1.55 ^c^	2.02 ^b^	2.61 ^a^	0.124	0.001
Magnesium	2.41 ^b^	3.93 ^a^	3.36 ^a^	0.205	0.005
Lactation 28 d, mmol/L					
Potassium	2.46	2.55	2.41	0.128	0.900
Magnesium	3.80	4.06	3.66	0.115	0.362

^a–c^ The values with different superscripts are significantly different at *p* < 0.05. ^1^ CON: sows were fed with a corn–soybean meal basal diet; PM: sows were fed with a basal diet supplemented with 0.20% magnesium sulfate and 0.15% potassium chloride; PMS: sows were fed with a basal diet supplemented with 0.45% natural potassium-magnesium sulfate.

**Table 6:** Effects of maternal dietary supplementation with two types of potassium and magnesium on the composition of colostrum and milk of sows (n = 15).

Items	Treatments ^1^			SEM	*p*-Value
	CON	PM	PMS		
Colostrum					
Fat, %	5.31	4.72	4.11	0.300	0.265
Protein, %	16.05	15.82	15.17	0.379	0.651
Lactose, %	1.98	2.00	2.18	0.107	0.754
Total solids, %	24.24	23.44	22.36	0.493	0.304
Solids-not-fat, %	18.93	18.72	18.24	0.293	0.652
Milk					
Fat, %	7.43	7.51	6.99	0.242	0.646
Protein, %	4.39	4.49	4.45	0.049	0.720
Lactose, %	5.75	5.71	5.82	0.061	0.775
Total solids, %	18.46	18.61	18.15	0.210	0.664
Solids-not-fat, %	11.04	11.10	11.17	0.065	0.748

^1^ CON: sows were fed with a corn–soybean meal basal diet; PM: sows were fed with a basal diet supplemented with 0.20% magnesium sulfate and 0.15% potassium chloride; PMS: sows were fed with a basal diet supplemented with 0.45% natural potassium-magnesium sulfate.

## Data Availability

The data will be available on request from corresponding authors.
